# Correspondence, Intelligence, &c.

**Published:** 1824-06-01

**Authors:** 


### INTELLIGENCE, CORRESPONDENCE, &c.

The analysis of Dr. Shearman's work on debility came safe to hand, from N. It was too late for this number; it will certainly appear in our next.

The queries of a "young surgeon" respecting wounded arteries will be answered in our next.

Many thanks to ÆQUUS for his medico-legal communication. His letter is under consideration.

Mr. Butler, herbalist in Covent Garden, has lately imported a small quantity of the secale cornutum, (ergot of rye,) a specimen of which he has obligingly sent us. A trial may now be made of this curious medicinal substance by our obstetric brethren.

### TYRO'S LETTER.

"SIR,—Will you have the goodness, in your next number, to explain how the tongue becomes furred, or to point out any author who explains the subject?

TYRO.

We consider the furring of the tongue as a morbid alteration in its glandular and papillary structure, or, at all events, a *morbid secretion* from its own surface, sympathetic of disorder in some other part of the body—generally of the stomach or bowels. "Toutes les fois," says Bichat, "qu'il y a embarras gastrique, la surface de l'estomac souffre, *par consequent la surface de la langue*; les glandes sur cette surface augmentent leur action, et de la cet enduit blanchatre et muqueux qui constitue ce que l'on appelle vulgairement *langue*

*chargée*, qui offre un veritable *catarrhe sympathique*." We believe this is as good an explanation as can be given; but if Tyro wishes for longer dissertations on the subject, he may consult the "SEMEIOLOGIE" of M. Double—the "Traité du Prògnostic" of Leroy—or last, not least, to Dr. Marshall Hall, on Diagnosis, p. 54, *et seq.*

---

### *Mr. Green's Lectures at the College of Surgeons.*

Mr. Green's lectures at the College attracted a most crowded audience, and called forth enthusiastic plaudits, this year. The subject was highly favourable—being no less than a delineation of the structure, functions, and natural history of the whole of animated nature, from the minutest animalcula up to man himself. The present course ascended to the conclusion of the invertebrated animals. Mr. Green not only displayed an intimate acquaintance with every link in this immense chain, but elucidated each subject by means of beautiful magnified drawings, diagrams, and figures, in aid of the costly and numerous preparations preserved in the College Museum. Nothing could exceed the *matter* thus brought forward, except the *manner* in which it was delivered. The most abstruse points of physiology and anatomy were descanted on in an easy and flowing oratory that would have done honour to the senate or the bar. In fine, we have no hesitation in averring that, in point of elocution, Mr. Green is the first medical lecturer we have ever heard in this or in any other conntry.

---

### MEDICATED OIL SILK.

Mr. Morange, from New York, has established an extensive manufactury of medicated oil silk, in Middlesex Terrace, Hackney Road. We have had letters from several distinguished physicians in the United States, strongly recommending this manufacture, in cases of CHRONIC RHEUMATISM and GOUT, and we have seen documents to the same effect from Dr. Mitchell, Dr. Post, Dr. Hosack, Dr. Watts, Dr. Stevens, Dr. Pascalis, and several others. With such respectable vouchers, we have no doubt that Mr. Morange will meet with due encouragement in this metropolis.

---

### LECTURES ANNOUNCED.

Dr. Ramadge F.L.S. Fellow of the Royal College of Physicians, and Physician to the Infirmary for Diseases of the Lungs, &c. will commence his Summer Course of Lectures and Examinations on the Principles and Practice of Physic, Materia Medica, and General and Pharmaceutic Chemistry, at a quarter past Ten in the Morning of Monday, the 7th of June. Three Courses are delivered every Year; and for particulars apply to Dr. Ramadge, at his house, 21, Ely Place.

Dr. Dunglison, Consulting Physician-Accoucheur to the Eastern Dispensary, will commence a Course of Lectures on the Principles and Practice of Midwifery, &c. at the commencement of October next.

Dr. Copland will commence, about the end of September next, Courses of Lectures on the Principles of Pathology and the Practice of Medicine, and on the Materia Medica.

---

### FOTHERGILLIAN MEDAL.

We are most happy to see the Fothergillian Medal, value 20 guineas, awarded to Mr. Bampfield, of Bedford Street, Covent Garden, by the Medical Society of London, for the best Treatise on Spinal Affections· We hope soon to see the subject laid before the public at large by the able and indefatigable author.

### *Army Medical Officers' Supplemental and Benevolent Fund Dinner, Ninth Anniversary.*

The anniversary of this most excellent and praise-worthy institution was celebrated on the 14th May, at the Thatched House Tavern, with great harmony, conviviality, and decorum. Sir James Fellowes was in the chair, supported on right and left by Sir Henry Halford, Mr. Cline, Dr. Cooke, Sir Everard Home, and numerous other distinguished members of the profession. Upwards of 80 gentlemen sat down to an excellent dinner, and we observed an unusual proportion of visitors, from the other liberal and learned professions. After the routine toasts were disposed of, the *special* ones commenced, and drew forth numerous and neat speeches from the individuals on whom devolved the duty of returning thanks. The College of Physicians and Surgeons being toasted, thanks were returned in very appropriate and eloquent speeches, by Sir Henry Halford and Mr. Cline. The Worshipful Company of Apothecaries brought Mr. Tegart upon his legs—and the Royal Society called forth (we are bound to believe, for we could not hear it) the characteristic eloquence of Sir Everard Home. When the Church Militant (the Chaplains of the Army) was drunk, the Demosthenes of the party, the Rev. Mr. Clarke, delighted the company with an exquisite specimen of elocution, delivered in a very graceful and easy style. This brought forth thundering plaudits from all parts of the room. When the Medical Benevolent Fund was given, Sir Henry Halford again spoke with great effect. He eulogized the memory of his friend, and the profession's friend, Dr. Baillie, who had paid the debt of Nature since their last meeting, and complimented the present company on containing many who would imitate the example, and rival the fame, of the illustrious deceased. May this prediction be verified!

Success to the Naval Medical Supplemental Fund was given from the chair, and thanks returned by Dr. James Johnson. When the visitors' healths were given, several select and entertaining speeches were elicited. One, in particular, from an academician (Mr. Wilkie) attracted much attention. This gentleman drew an ingenious parallel between Medicine and Painting, always giving it in favour of the *former*. When, in the midst of his speech, he observed that—"*the painter might flatter the vanity, but it was the physician who could mitigate the sufferings of human nature;*" the house rung with applause, and interrupted, for some minutes, the orator. "Professor Brande and the Public Teachers of the Metropolis" called that distinguished lecturer upon his legs, amidst numerous symptoms of approbation. He confessed that the present honour done to him was quite unexpected, and therefore he had come to dinner *unprepared with a speech,* (much laughter.) The professor, however, returned thanks in a short and neat address, not at all the worse for being unpremeditated. We are unable to do justice to the various other orators on this festive occasion; but we cannot pass over the health of the ladies who had patronized the benevolent fund. Who was to return thanks? Mr. Guthrie, with his usual promptitude at wit and humour, got up and observed that, as the ladies had no representative at the dinner, he moved that one should be elected for that purpose, and proposed his opposite friend (Dr. George Gregory) as the *longest and the strongest* man in the company—and consequently the most proper champion for the ladies, (peals of laughter.) Dr. Gregory very goodnaturedly got up (somewhat above six feet, amid much merriment and applause) and proved that his eloquence was not inferior to his altitude on this occasion.

At half-past ten o'clock the chairman retired amidst loud plaudits—and Sir James M'Grigor was called to the vacant seat, with enthusiastic unanimity by all present. The evening concluded with great harmony and hilarity, and the guests separated after a material and intellectual banquet of no common kind. —*Perpetuum Esto.*—Hospes.

